# Supplementary material for: Antenatal Iron-Folic Acid Supplementation Is Associated with Improved Linear Growth and Reduced Risk of Stunting or Severe Stunting in South Asian Children Less than Two Years of Age: A Pooled Analysis from Seven Countries
Source: Nutrients. 2020 Aug 28;12(9):2632. doi: 10.3390/nu12092632 (PMC7551993; doi:10.3390/nu12092632)
Supplement: Supplementary file 1 [file nutrients-12-02632-s001.pdf]

Table S1: Effect of any iron-folic acid (IFA) supplementation, number of supplements used, the timing of the start of supplementation and a combination of timing of start with the number of supplements used on child stunting, severe stunting and LAZ in Afghanistan 2013 (n=6343): adjusted Poisson regression (Stunting & Severe stunting) and linear regression (LAZ)

	Stunting (LAZ<-2)				Severe stunting (LAZ<-3)				LAZ			
**Variables**	**aRR**	**95% CI**		**p**	**aRR**	**95% CI**		**p**	**Coefficients**	**95% CI**		**p**
**IFA supplements used**												
No	1.00	(reference)			1.00	(reference)			-	(reference)		
Yes	0.90	0.77	1.04	0.155	0.89	0.71	1.13	0.348	0.15	-0.04	0.34	0.130
**Number of IFA supplements used during pregnancy**												
No IFA used	1.00	(reference)			1.00	(reference)			-	(reference)		
< 120 IFA used	0.86	0.72	1.04	0.116	0.85	0.66	1.12	0.259	0.08	-0.11	0.28	0.398
≥ 120 IFA used	1.01	0.61	1.67	0.965	1.70	0.80	3.63	0.169	0.12	-0.73	0.96	0.790
**Timing of initiation of IFA supplements**												
No IFA used	1.00	(reference)			1.00	(reference)			-	(reference)		
Up to 4 months of pregnancy	0.90	0.73	1.11	0.323	0.98	0.73	1.32	0.911	0.04	-0.19	0.27	0.739
More than 4 months of pregnancy	0.80	0.62	1.05	0.104	0.83	0.54	1.27	0.393	0.21	-0.12	0.53	0.208
**Timing of initiation and number of IFA supplements used**												
No IFA used	1.00	(reference)			1.00	(reference)			-	(reference)		
Up to 4 months of pregnancy and <120 IFA used	0.92	0.73	1.15	0.458	0.93	0.67	1.28	0.650	-0.02	-0.25	0.22	0.893
Up to 4 months of pregnancy and ≥ 120 IFA used	0.91	0.52	1.60	0.737	1.81	0.87	3.76	0.112	0.14	-0.73	1.00	0.760
More than 4 months of pregnancy and any IFA used	0.77	0.60	1.00	0.051	0.72	0.50	1.04	0.080	0.29	0.03	0.56	0.031

Adjusted for region, area of residence, maternal marital status, maternal educational status, fuel used for cooking, source of drinking water, sanitation facilities, pooled household wealth index, maternal age at childbirth, sex of child, the timing of initiation of breastfeeding, age of the child, and child had diarrhea during last two weeks before the interview. Also, we adjusted the model for the duration of recall. We excluded 52 missing records from the analysis.

aRR: Adjusted relative risk.

CI: Confidence interval.

IFA: Iron/folic acid.

Height-for-Age Z-score

Table S2: Effect of any iron-folic acid (IFA) supplementation, and timing of the start of supplementation on child stunting, severe stunting and LAZ in Bangladesh 2007 (n=2079): adjusted Poisson regression (Stunting & Severe stunting) and linear regression (LAZ)

	Stunting (LAZ<-2)				Severe stunting (LAZ<-3)				LAZ			
**Variables**	**aRR**	**95% CI**		**p**	**aRR**	**95% CI**		**p**	**Coefficients**	**95% CI**		**p**
**IFA supplements used**												
No	1.00	(reference)			1.00	(reference)			-	(reference)		
Yes	0.89	0.74	1.06	0.178	0.86	0.59	1.25	0.435	0.13	0.02	0.25	0.019
**Timing of initiation of IFA supplements**												
No IFA used	1.00	(reference)			1.00	(reference)			-	(reference)		
Up to 4 months of pregnancy	0.84	0.65	1.07	0.162	0.99	0.59	1.65	0.960	0.28	0.12	0.43	<0.0001
More than 4 months of pregnancy	0.85	0.68	1.06	0.152	0.84	0.65	1.02	0.138	0.13	-0.01	0.27	0.066

Adjusted for region, area of residence, maternal marital status, maternal educational status, fuel used for cooking, source of drinking water, sanitation facilities, pooled household wealth index, maternal age at childbirth, sex of child, the timing of initiation of breastfeeding, age of the child, and child had diarrhea during last two weeks before the interview. Also, we adjusted the model for the duration of recall. Four missing records were excluded from the analysis. The survey did not collect information on the number of IFA supplements used.

aRR: Adjusted relative risk.

CI: Confidence interval.

IFA: Iron/folic acid.

Height-for-Age Z-score

Table S3: Effect of any iron-folic acid (IFA) supplementation, and timing of the start of supplementation on child stunting, severe stunting and LAZ in Bhutan 2015 (n=562): adjusted Poisson regression (Stunting & Severe stunting) and linear regression (LAZ)

	Stunting (LAZ<-2)				Severe stunting (LAZ<-3)				LAZ			
**Variables**	**aRR**	**95% CI**		**p**	**aRR**	**95% CI**		**p-value**	**Coefficients**	**95% CI**		**p**
**IFA supplements used**												
No	1.00	(reference)			1.00	(reference)			-	(reference)		
Yes	0.91	0.16	5.04	0.911	1.42	0.14	13.89	0.764	0.89	0.17	1.61	0.015
**Timing of initiation of IFA supplements**												
No IFA used	1.00	(reference)			1.00	(reference)			-	(reference)		
Up to 4 months of pregnancy	0.85	0.15	4.8	0.857	1.40	0.14	14.06	0.772	0.87	0.14	1.61	0.021
More than 4 months of pregnancy	1.16	0.2	6.65	0.864	1.47	0.13	17.07	0.758	0.98	0.2	1.77	0.015

Adjusted for region, area of residence, maternal marital status, maternal educational status, fuel used for cooking, source of drinking water, sanitation facilities, pooled household wealth index, maternal age at childbirth, sex of child, the timing of initiation of breastfeeding, age of the child, and child had diarrhea during last two weeks before the interview. Also, we adjusted the model for the duration of recall. We excluded 25 missing records from the analysis. The survey did not collect information on the number of IFA supplements used.

aRR: Adjusted relative risk.

CI: Confidence interval.

IFA: Iron/folic acid.

Height-for-Age Z-score

Table S4: Effect of any iron-folic acid (IFA) supplementation, number of supplements used, the timing of the start of supplementation and a combination of timing of start with the number of supplements used on child stunting, severe stunting and LAZ in India 2016 (n=83188), adjusted Poisson regression

	Stunting (LAZ<-2)				Severe stunting (LAZ<-3)				LAZ			
**Variables**	**aRR**	**95% CI**		**p**	**aRR**	**95% CI**		**p**	**Coefficients**	**95% CI**		**p**
**IFA used**												
No	1.00	(reference)			1.00	(reference)			-	(reference)		
Yes	0.95	0.92	0.99	0.006	0.93	0.88	0.98	0.017	0.06	0.02	0.09	0.001
**Number of IFA used during pregnancy**												
No IFA used	1.00	(reference)			1.00	(reference)			-	(reference)		
< 120 IFA used	0.96	0.92	0.99	0.014	0.92	0.86	0.98	0.010	0.05	0.02	0.09	0.004
≥ 120 IFA used	0.90	0.84	0.96	0.001	0.88	0.79	0.98	0.029	0.08	0.02	0.14	0.008
**Timing of initiation of IFA supplements**												
No IFA used	1.00	(reference)			1.00	(reference)			-	(reference)		
Up to 4 months of pregnancy	0.94	0.91	0.97	0.001	0.90	0.84	0.96	0.001	0.07	0.03	0.10	<0.0001
More than 4 months of pregnancy	0.95	0.91	1.01	0.083	0.95	0.86	1.05	0.322	0.04	-0.01	0.09	0.139
**Timing of initiation and number of IFA used**												
No IFA used	1.00	(reference)			1.00	(reference)			-	(reference)		
Up to 4 months of pregnancy and <120 IFA used	0.95	0.92	0.99	0.010	0.92	0.86	0.98	0.011	0.06	0.02	0.10	0.003
Up to 4 months of pregnancy and ≥ 120 IFA used	0.90	0.85	0.96	0.002	0.90	0.80	1.00	0.058	0.08	0.02	0.14	0.010
More than 4 months of pregnancy and any IFA used	0.97	0.91	1.03	0.278	0.91	0.81	1.02	0.098	0.04	-0.02	0.95	0.229

Adjusted for geographic region, area of residence, maternal marital status, maternal educational status, fuel used for cooking, source of drinking water, sanitation facilities, household wealth index, maternal age at childbirth, sex of child, the timing of initiation of breastfeeding, age of the child, and child had diarrhea during last two weeks before the interview. Also, we adjusted the model for the duration of recall for the model for stunting. We excluded 384 missing records from the analysis.

aRR: Adjusted relative risk.

CI: Confidence interval.

IFA: Iron/folic acid.

Height-for-Age Z-score

Table S5: Effect of any iron-folic acid (IFA) supplementation, number of supplements used, the timing of the start of supplements and a combination of timing of start with the number of supplements used on child stunting, severe stunting and LAZ in Maldives 2009 (n=992): adjusted Poisson regression (Stunting & Severe stunting) and linear regression (LAZ)

	Stunting (LAZ<-2)				Severe stunting (LAZ<-3)				LAZ			
**Variables**	**aRR**	**95% CI**		**p**	**aRR**	**95% CI**		**p**	**Coefficients**	**95% CI**		**p**
**IFA supplements used**												
No	1.00	(reference)			1.00	(reference)			-	(reference)		
Yes	0.89	0.59	1.33	0.564	0.94	0.31	2.8	0.906	0.18	-0.13	0.49	0.249
**Number of IFA supplements used during pregnancy**												
No IFA used	1.00	(reference)			1.00	(reference)			-	(reference)		
< 120 IFA used	0.61	0.32	1.18	0.145	0.74	0.2	2.72	0.651	0.21	-0.21	0.63	0.333
≥120 IFA used	1.00	0.62	1.63	0.995	0.73	0.23	2.31	0.595	0.22	-0.13	0.57	0.211
**Timing of initiation of IFA supplements**												
No IFA used	1.00	(reference)			1.00	(reference)			-	(reference)		
Up to 4 months of pregnancy	0.9	0.60	1.30	0.466	1.32	0.31	5.65	0.703	-0.19	-0.68	0.31	0.457
More than 4 months of pregnancy	1.3	0.60	2.70	0.493	0.85	0.28	2.52	0.765	0.19	-0.11	0.5	0.217
**Timing of initiation and number of IFA supplements used**												
No IFA used	1.00	(reference)			1.00	(reference)			-	(reference)		
Up to 4 months of pregnancy and <120 IFA used	0.62	0.27	1.42	0.254	0.60	0.15	2.37	0.465	0.26	-0.17	0.69	0.242
Up to 4 months of pregnancy and ≥ 120 IFA used	0.96	0.54	1.70	0.887	0.76	0.24	2.39	0.635	0.22	-0.12	0.57	0.205
More than 4 months of pregnancy and any IFA used	2.15	1.04	4.41	0.038	1.39	0.22	8.79	0.725	-0.46	-1.17	0.25	0.203

Adjusted for region, area of residence, maternal marital status, maternal educational status, fuel used for cooking, source of drinking water, sanitation facilities, pooled household wealth index, maternal age at childbirth, sex of child, the timing of initiation of breastfeeding, age of the child, and child had diarrhea during last two weeks before the interview. Also, we adjusted the model for the duration of recall. We excluded 32 missing records w from the analysis.

aRR: Adjusted relative risk.

CI: Confidence interval.

IFA: Iron/folic acid.

Height-for-Age Z-score

Table S6: Effect of any iron-folic acid (IFA) supplementation, number of supplements used, the timing of the start of supplements and a combination of timing of start with the number of supplements used on child stunting, severe stunting and LAZ in Nepal 2006-07 (n=1944): adjusted Poisson regression (Stunting & Severe stunting) and linear regression (LAZ)

	Stunting (LAZ<-2)				Severe stunting (LAZ<-3)				LAZ			
**Variables**	**aRR**	**95% CI**		**p**	**aRR**	**95% CI**		**p**	**Coefficients**	**95% CI**		**p**
**IFA supplements used**												
No	1.00	(reference)			1.00	(reference)			1.00	(reference)		
Yes	0.83	0.70	0.98	0.027	0.47	0.33	0.68	<0.0001	0.24	0.09	0.40	0.002
**Number of IFA supplements used during pregnancy**												
No IFA used	1.00	(reference)			1.00	(reference)			1.00	(reference)		
< 120 IFA used	0.84	0.70	1.02	0.080	0.50	0.33	0.76	0.001	0.20	0.03	0.37	0.019
120 and more IFA used	0.68	0.49	0.96	0.029	0.29	0.14	0.59	0.001	0.45	0.23	0.66	<0.0001
**Timing of initiation of IFA supplements**												
No IFA used	1.00	(reference)			1.00	(reference)			1.00	(reference)		
Up to 4 months of pregnancy	0.86	0.70	1.06	0.16	0.50	0.30	0.84	0.008	0.24	0.05	0.43	0.013
More than 4 months of pregnancy	0.80	0.63	1.00	0.052	0.46	0.30	0.70	<0.0001	0.24	0.07	0.41	0.005
**Timing of initiation and number of IFA supplements used**												
No IFA used	1.00	(reference)			1.00	(reference)			1.00	(reference)		
Up to 4 months of pregnancy and <120 IFA used	0.96	0.76	1.20	0.694	0.60	0.34	1.05	0.073	0.119924	-0.09	0.33	0.251
Up to 4 months of pregnancy and 120 or more IFA used	0.67	0.45	0.99	0.045	0.27	0.12	0.64	0.003	0.449846	0.22	0.68	<0.0001
More than 4 months of pregnancy and any IFA used	0.75	0.60	0.95	0.017	0.41	0.26	0.65	<0.0001	0.289169	0.11	0.46	0.001

Adjusted for region, area of residence, maternal marital status, maternal educational status, fuel used for cooking, source of drinking water, sanitation facilities, pooled household wealth index, maternal age at childbirth, sex of child, the timing of initiation of breastfeeding, age of the child, and child had diarrhea during last two weeks before the interview. Also, we adjusted the model for the duration of recall. We excluded 15 missing records from the analysis.

aRR: Adjusted relative risk.

CI: Confidence interval.

IFA: Iron/folic acid.

Height-for-Age Z-score

Table S7: Effect of any iron-folic acid (IFA) supplementation, number of supplements used, the timing of the start of supplements and a combination of timing of start with the number of supplements used on child stunting, severe stunting and LAZ in Pakistan 2012-13: adjusted Poisson regression (Stunting & Severe stunting) and linear regression (LAZ)

	Stunting (LAZ<-2)				Severe stunting (LAZ<-3)				LAZ			
**Variables**	**aRR**	**95% CI**		**p**	**aRR**	**95% CI**		**p**	**Coefficients**	**95% CI**		**p**
**IFA supplements used**												
No	1.00	(reference)			1.00	(reference)			1.00	(reference)		
Yes	0.59	0.45	0.78	<0.0001	0.94	0.65	1.37	0.742	0.38	0.14	0.62	0.002
**Number of IFA supplements used during pregnancy**												
No IFA used	1.00	(reference)			1.00	(reference)			1.00	(reference)		
< 120 IFA used	0.52	0.37	0.72	<0.0001	0.78	0.49	1.24	0.292	0.52	0.24	0.79	<0.000 1
120 and more IFA used	0.54	0.33	0.91	<0.0001	1.03	0.52	2.04	0.927	0.39	0.01	0.77	0.047
**Timing of initiation of IFA supplements**												
No IFA used	1.00	(reference)			1.00	(reference)			1.00	(reference)		
Up to 4 months of pregnancy	0.45	0.31	0.65	<0.0001	0.74	0.46	1.2	0.224	0.61	0.35	0.87	<0.000 1
More than 4 months of pregnancy	0.70	0.47	1.04	0.08	1.16	0.63	2.13	0.64	0.09	-0.26	0.43	0.620
**Timing of initiation and number of IFA supplements used**												
No IFA used	1.00	(reference)			1.00	(reference)			1.00	(reference)		
Up to 4 months of pregnancy and <120 IFA used	0.42	0.27	0.64	0	0.71	0.41	1.24	0.226	0.69	0.39	0.99	<0.000 1
Up to 4 months of pregnancy and 120 or more IFA used	0.53	0.31	0.9	0.019	0.93	0.44	1.95	0.85	0.46	0.07	0.84	0.019
More than 4 months of pregnancy and any IFA used	0.71	0.46	1.1	0.128	0.99	0.52	1.87	0.974	0.17	-0.23	0.57	0.407

Adjusted for region, area of residence, maternal marital status, maternal educational status, fuel used for cooking, source of drinking water, sanitation facilities, pooled household wealth index, maternal age at childbirth, sex of child, the timing of initiation of breastfeeding, age of the child, and child had diarrhea during last two weeks before the interview. Also, we adjusted the model for the duration of recall. We excluded 16 missing records from the analysis.

aRR: Adjusted relative risk.

CI: Confidence interval.

IFA: Iron/folic acid.

Height-for-Age Z-score

Table S8: Factors associated with stunting, severe stunting and LAZ in four South Asian countries (n=86103): Adjusted Poisson regression (Stunting and Severe stunting) and linear regression (LAZ)

	Stunting (LAZ<-2)				Severe stunting (LAZ<-3)				LAZ			
**Factors**	**aRR**	**95% CI**		**p**	**aRR**	**95% CI**		**p**	**Coefficient**	**95% CI**		**p**
**Community-level and socio-economic status factors**												
**Country**												
India	1.00	(reference)			1.00	(reference)			1.00	(reference)		
Maldives	0.89	0.76	1.05	0.168	0.71	0.51	0.99	0.047	-0.12	-0.23	-0.01	0.042
Nepal	0.84	0.76	0.93	0.001	0.62	0.51	0.76	<0.0001	-0.01	-0.10	0.08	0.838
Pakistan	1.09	0.97	1.23	0.152	1.21	0.97	1.50	0.091	-0.16	-0.27	-0.05	0.006
**Fuel used for cooking**												
Biomass energy	1.00	(reference)			1.00	(reference)			1.00	(reference)		
Natural gas	0.87	0.83	0.91	<0.0001	0.84	0.78	0.91	<0.0001	0.17	0.13	0.21	<0.0001
**Maternal educational status**												
Secondary and above	1.00	(reference)			1.00	(reference)			1.00	(reference)		
Up to primary	1.19	1.14	1.25	<0.0001	1.25	1.15	1.35	<0.0001	-0.19	-0.23	-0.14	<0.0001
No education	1.31	1.27	1.36	<0.0001	1.47	1.38	1.57	<0.0001	-0.30	-0.33	-0.26	<0.0001
**Source of drinking water**												
Improved	1.00	(reference)			1.00	(reference)			1.00	(reference)		
Unimproved	0.94	0.89	0.98	0.011	0.88	0.80	0.96	0.005	0.07	0.03	0.12	0.003
**Sanitary Facility**												
Improved	1.00	(reference)			1.00	(reference)			1.00	(reference)		
Unimproved	1.12	1.07	1.17	<0.0001	1.11	1.03	1.20	0.007	-0.08	-0.13	-0.04	<0.0001
**Household wealth index**												
Quintile 1 (Wealthiest)	1.00	(reference)			1.00	(reference)			1.00	(reference)		
Quintile 2	1.34	1.05	1.22	0.001	1.18	1.04	1.35	0.012	-0.11	-0.17	-0.06	<0.0001
Quintile 3 (Middle)	1.17	1.08	1.26	<0.0001	1.25	1.09	1.43	0.001	-0.18	-0.24	-0.11	<0.0001
Quintile 4	1.23	1.13	1.33	<0.0001	1.36	1.19	1.57	<0.0001	-0.25	-0.32	-0.18	<0.0001
Quintile 5 (Poorest)	1.25	1.15	1.36	<0.0001	1.54	1.34	1.78	<0.0001	-0.30	-0.37	-0.22	<0.0001
**Maternal and child characteristics**												
**Maternal age at childbirth**												
20-24 years	1.00	(reference)			NS				1.00	(reference)		
< 20 years	1.02	0.99	1.05	0.171					-0.04	-0.07	-0.01	0.013
>24 years	0.89	0.85	0.94	<0.0001					0.14	0.09	0.19	<0.0001
**Sex of child**												
Female	1.00	(reference)			1.00	(reference)			1.00	(reference)		
Male	1.08	1.05	1.11	<0.0001	1.16	1.10	1.22	<0.0001	-0.12	-0.15	-0.09	<0.0001
**Child diarrhea during the last two weeks before the interview**												

No					NS				1.00	(reference)		
Yes									-0.04	-0.07	0.00	0.058
**Maternal perceived birth size**												
Average or larger than average	1.00	(reference)			1.00	(reference)			1.00	(reference)		
Smaller than average	1.31	1.27	1.36	<0.0001	1.41	1.32	1.51	<0.0001	-0.36	-0.40	-0.31	<0.0001
**Perinatal health services variable**												
**Number of ANC visits**												
No ANC visit	1.00	(reference)			1.00	(reference)			1.00	(reference)		
1-3 ANC visits	0.95	0.92	0.99	0.005	0.91	0.85	0.96	0.002	0.01	-0.03	0.06	0.555
4 or more ANC visits	0.86	0.83	0.90	<0.0001	0.81	0.76	0.87	<0.0001	0.13	0.08	0.17	<0.0001

Adjusted for country, area of residence, maternal marital status, maternal educational status, fuel used for cooking, source of drinking water, sanitation facilities, pooled household wealth index, maternal age at childbirth, sex of child, the timing of initiation of breastfeeding, age of the child, and child had diarrhea during last two weeks before the interview. Also, we adjusted the model for the duration of recall and maternal perceived birth size. We excluded 89 missing records from the analysis.

aRR: Adjusted relative risk.

CI: Confidence interval.

IFA: Iron/folic acid.

Height-for-Age Z-score

NS: Not significant

SD: Standard deviation

Table S9: Effect of any iron-folic acid (IFA) supplementation, number of supplements used, the timing of the start of supplements and a combination of timing of start with the number of supplements used on child stunting, severe stunting and LAZ in four South Asian countries (n=86103): adjusted Poisson regression (Stunting & Severe stunting) and linear regression (LAZ)

	Stunting (LAZ<-2)				Severe stunting (LAZ<-3)				LAZ			
**Variables**	**aRR**	**95% CI**		**p**	**aRR**	**95% CI**		**p**	**Coefficients**	**95% CI**		**p**
**IFA supplements used**												
No	1.00	(reference)			1.00	(reference)			1.00	(reference)		
Yes	0.93	0.90	0.96	<0.0001	0.93	0.88	0.98	0.018	0.09	0.05	0.12	<0.0001
**Number of IFA supplements used during pregnancy**												
No IFA used	1.00	(reference)			1.00	(reference)			1.00	(reference)		
< 120 IFA used	0.93	0.90	0.97	<0.0001	0.92	0.87	0.98	0.009	0.08	0.04	0.11	<0.0001
120 and more IFA used	0.87	0.82	0.92	<0.0001	0.89	0.81	0.99	0.039	0.14	0.09	0.20	<0.0001
**Timing of initiation of IFA supplements**												
No IFA used	1.00	(reference)			1.00	(reference)			1.00	(reference)		
Up to 4 months of pregnancy	0.92	0.88	0.95	<0.0001	0.90	0.85	0.96	0.001	0.10	0.07	0.14	<0.0001
More than 4 months of pregnancy	0.93	0.89	0.98	0.012	0.97	0.88	1.07	0.563	0.06	0.01	0.12	0.017
**Timing of initiation and number of IFA supplements used**												
No IFA used	1.00	(reference)			1.00	(reference)			1.00	(reference)		
Up to 4 months of pregnancy and <120 IFA us	0.93	0.89	0.96	<0.0001	0.91	0.86	0.97	0.006	0.08	0.05	0.12	<0.0001
Up to 4 months of pregnancy and 120 or more IFA used ed	0.87	0.82	0.93	<0.0001	0.91	0.81	1.00	0.070	0.14	0.09	0.20	<0.0001
More than 4 months of pregnancy and any IFA used	0.95	0.90	1.00	0.088	0.94	0.84	1.04	0.238	0.05	-0.01	0.10	0.114

Adjusted for country, area of residence, maternal marital status, maternal educational status, fuel used for cooking, source of drinking water, sanitation facilities, pooled household wealth index, maternal age at childbirth, sex of child, the timing of initiation of breastfeeding, age of the child, and child had diarrhea during last two weeks before the interview. Also, we adjusted the model for the duration of recall and maternal perceived birth size. We excluded 89 missing values from the analysis.

aRR: Adjusted relative risk.

CI: Confidence interval.

IFA: Iron/folic acid.

Height-for-Age Z-score
